# Evaluation of the sublethal effect of tetrachlorantraniliprole on *Spodoptera exigua* and its potential toxicity to two non-target organisms

**DOI:** 10.1371/journal.pone.0242052

**Published:** 2020-11-09

**Authors:** Haiyuan Teng, Yongda Yuan, Tianshu Zhang, Xiaoli Chang, Dongsheng Wang

**Affiliations:** Institute of Eco-Environmental and Plant Protection, Shanghai Academy of Agricultural Sciences, Shanghai, China; Institut Sophia Agrobiotech, FRANCE

## Abstract

Tetrachlorantraniliprole (TCAP) is a novel anthranilic diamide insecticide that specifically targets the ryanodine receptors of lepidopteran insect species with excellent insecticidal activity. Previous studies have reported the sublethal effects of multiple diamides on several lepidopteran species, whereas the sublethal and non-target effects of TCAP remain largely unknown. We assessed the sublethal effects of TCAP on *Spodoptera exigua*. We also investigated the effects of TCAP on non-target *Harmonia axyridis* and *Eisenia fetida*, *S*. *exigua* was more sensitive to TCAP than to chlorantraniliprole, as the LC_50_ (10.371 μg L^-1^ at 72 h) of TCAP was relatively lower. Compared with those of the control, sublethal concentrations of TCAP (LC_10_ and LC_30_) not only prolonged the duration of the larval and pupal stages as well as the mean generation time but also reduced certain population parameters. On the other hand, TCAP exposure, even at the highest concentration, did not induce toxic effects in *H*. *axyridis* ladybugs (1^st^ instar larvae and adults) or *E*. *fetida* earthworms. Taken together, our results suggest that TCAP can be used as a novel and promising component of the integrated pest management (IPM) program against *S*. *exigua* due to its robust target effects and negligible non-target risks.

## Introduction

*Spodoptera exigua* (Hübner) (Lepidoptera: Noctuidae) is a notorious insect pest that causes devastating damage to various vegetables and field crops, such as tomatoes, cowpeas, corn, eggplant, peppers, watermelon, and soybeans [1]. Insecticides have been the conventional methods used for the control of this species; however, in addition to its evolved resistance to traditional insecticides (organophosphates, carbamates, and pyrethroids) [2], emerging resistance of *S*. *exigua* to multiple novel pesticides (e.g., spinosad, abamectin, indoxacarb, and tebufenozide) is also documented [3], thus resulting in failed control efforts. Additionally, the overuse and indiscriminate application of these insecticides chemicals may pose a severe threat to a variety of beneficial organisms, including natural enemies, parasites, soil microbes, earthworms, etc. [4, 5]. Sublethal effects refer to physiological and/or behavioral changes in the surviving individuals following exposure to an insecticide at sublethal doses [6, 7]. In terms of pest management, therefore, insecticides should be selectively employed, taking their physicochemical properties, mode of action and toxicity against non-target species into account [8].

Diamides are a novel class of insecticides used in vegetable production for the control of lepidopteran pests. These insecticides bind to the ryanodine receptor (RyR) and activate the calcium channel, causing an excessive release of intracellular calcium ions, muscle contraction failure and eventual insect death [9]. They are categorized into Group 28 by the Insecticide Resistance Action Committee [10]. A growing body of evidence has revealed the sublethal effects of diamide insecticides on the development and reproduction of *Spodoptera litura* [11], *Helicoverpa assulta* [12], *Agrotis ipsilon* [13], *S*. *exigua* [14], and *Plutella xylostella* [15]. Although tetrachlorantraniliprole (TCAP) has high insecticidal activity against *S*. *exigua* [16, 17], little information is available regarding the sublethal effects of TCAP, and its potential toxicological effects on non-target organisms are also poorly understood.

The objectives of this study were to (i) investigate the sublethal effects of TCAP on the life table parameters of *S*. *exigua* and (ii) assess the acute toxicity of TCAP to *Harmonia axyridis* (Coleoptera: Coccinellidae) and *Eisenia fetida* (Annelida: Lumbricidae).

## Materials and methods

### Ethics statement

This experiment did not involve any endangered or protected species. The earthworms were purchased from the College of Animal Sciences, Zhejiang University, China.

### Biological materials

The *S*. *exigua* larvae used in this study were collected in August 2016 from *Allium fistulosum* L. at Wusi Farm, Fengxian district Shanghai, China. The first (1^st^) instar larvae were transferred into 24-well plates and fed an artificial diet [18]. Pupae of *S*. *exigua* were placed in a 200-mL plastic cup until eclosion. The emerged adults were transferred into 100-mesh cages (20 cm in length, 20 cm in width, and 20 cm in height), with 50 female and 50 male adults per cage and were fed a honey-based solution (10%). Subsequently, the eggs were collected on A4 paper. The conditions throughout the *S*. *exigua* rearing procedures were 27 ± 1°C and 60 ± 5% relative humidity (RH) with a photoperiod of 14:10 h (L:D).

We used *Aphis glycines* (Matsumura) purchased from Beijing Kuoye Tianyuan Biotechnology Co., Ltd, China, to rear *H*. *axyridis* (Pallas). The colony of *A*. *glycines* was collected from a soybean field in May 2014 and was maintained on fava beans (*Vicia faba* L.), while *H*. *axyridis* adults were reared in 100-mesh cages (20 cm length, 20 cm width, 20 cm height) containing plants infested with *A*. *glycines*. All the tested insects were maintained under the following conditions: 25 ± 1°C, 70 ± 5% RH and a 16:8 h (L: D) photoperiod.

Healthy adult worms of *E*. *fetida* (Savigny) (weighing 360–410 mg) with well-developed clitella, an obvious girdle and uniform size was purchased from the College of Animal Sciences, Zhejiang University, China, and maintained in artificial soil at 20 ± 1°C under dark conditions in a climate chamber. The artificial soil consisted of 10% ground sphagnum peat (<0.5 mm), 20% kaolinite clay (>50% kaolinite), and 70% fine sand [19, 20]. A small amount of calcium carbonate was added to adjust the pH to 6.0 ± 0.5. Distilled water was replenished on a weekly basis to maintain the maximum water-holding capacity of the soil at approximately 35%.

### Insecticides

Formulated insecticides were used in all bioassays. The TCAP used at 100 g L^-1^ SC (9080^TM^ 100 SC) in this study was provided by Shenyang Kechuang Chemical Co., Ltd., Shenyang, China. The lambda-cyhalothrin used at 250 g L^-1^ EW (Kung Fu^®^ 25 EW) in this study was obtained from Syngenta Nantong Crop Protection Co., Ltd., Nantong, China.

### Target toxicity to *S*. *exigua*

The toxicity of TCAP to the first (1^st^) and third (3^rd^) instar larvae of *S*. *exigua* was assessed by IRAC method no. 20 [21], viz., through oral delivery. Five-to-nine concentrations of TCAP were prepared as serial dilutions with distilled water. A quantity of 100 mL of each concentration of diluted TCAP was mixed thoroughly with 25 g of artificial diet in a 500-mL plastic box. The artificial diet mixed with distilled water was used for the control group. Each group contained 24-well plates (3 mL per well with 2 mL of artificial diet), and one 1^st^ instar (newly hatched larvae<12 h) was deposited into each well. All the groups were maintained in an incubator at 27 ± 1°C and 60 ± 5% relative humidity (RH) with a photoperiod of 14:10 h (L:D). The mortality was recorded after incubation for 72 h. Larvae that did not respond to a brush touch were considered dead [22].

### Sublethal effects on biological and population parameters

In this experiment, life table theory was used to evaluate the sublethal effects of TCAP on *S*. *exigua*. An age-stage, two-sex life table was established to assess the population growth [23]. First, approximately 400 eggs laid on the same day were collected and cultured. When most of the eggs had developed into 1^st^ instar larvae, 300 larvae were selected for the life table study. Based on the prior acute toxicity test, newly molted 1^st^ instar larvae were treated with sublethal concentrations of TCAP (LC_10_, 0.578 μg a.i. L^-1^; LC_30_, 3.182 μg a.i. L^-1^) or distilled water (control). More specifically, one 1^st^ instar larva and approximately 2 mL of artificial diet containing LC_10_, LC_30_ or distilled water were placed in each well of the 24-well plates, with 100 1^st^ instar larvae per group. The developmental times and mortalities of all individuals were recorded until they reached the pupal stage. The weight of the pupae was measured at 24 h after pupation. The identification of pupal sex followed the instructions of Liu et al. [24].

Pupae of *S*. *exigua* were placed in plastic cups (7.3 and 5.2 cm top and bottom diameters, respectively, and 8.5 cm in height), and each plastic cup contained only one pupa. Pupae that failed to emerge as adults after 15 days were considered dead [12]. After hatching, an adult female and two adult males were paired in a plastic cup covered by a piece of black cloth for mating and oviposition. In addition, a folded piece of white paper of 7.0 cm in length was placed in the plastic cup to collect the eggs and replaced daily. Honey-based solution (10%) was provided as food to the couples every day until the female insect died. The eggs on the paper were counted daily, and the life spans of the pupae and adults were recorded. During the experiment, the dead individuals were removed and not replenished. All *S*. *exigua* individuals in different developmental stages were incubated under the same conditions described above.

### Non-target toxicity to *H*. *axyridis*

Using the method described by He et al. [25], 3^rd^ instar larvae and adults (2 days old) of the ladybug *H*. *axyridis* were exposed to insecticides in this assay. The TCAP concentration was applied according to a geometric series (2, 1, 0.5 and 0.25 mL L^−1^). The insecticide lambda-cyhalothrin (12.5 mg L^−1^) was applied at the recommended rate against lepidoptera, i.e., 0.05% (v/v), which was equivalent to 0.00125% (12.5 mg L^−1^) (w/v) of lambda-cyhalothrin, as the active substance.

To evaluate the acute toxicity of TCAP toward *H*. *axyridis* larvae and adults, petri dishes (9 cm in diameter) lined with 9-cm filter paper were prepared, and then a 1 mL solution of insecticide was applied to the center of the filter paper disks using an eppendorf pipette (Eppendorf, Hamburg, Germany), with distilled water as the control. The filter paper disks were allowed to dry for 30 min at room temperature before 3^rd^ instar larvae or adults were transferred to the petri dishes. The individuals of *H*. *axyridis* were moved to petri dishes and fed *A*. *glycines*. Ten insects were tested per replicate and four replicates (N = 4) were used per tested concentration. The insects were maintained at 25 ± 1°C, 70 ± 5% RH, and a 16:8 h (L:D). The mortality was recorded after 48 h.

### Non-target toxicity to *E*. *fetida*

The standard OECD [19] method was followed to test the toxicity of TCAP to *E*. *fetida* earthworms. In these assays, artificial soil was characterized by the same composition and pH as that described for *E*. *fetida* rearing; the soil was spiked with TCAP at concentrations of 1000, 500 and 250 mg kg^−1^. We used lambda-cyhalothrin at 32, 16 and 8 mg kg^−1^ of dry soil as the positive control, while distilled water was used as the negative control. The serial concentrations of TCAP and lambda-cyhalothrin were mixed into the soil (650 g). Ten earthworms were placed in a black plastic box (surface diameter, 18 cm; bottom diameter, 14 cm; height, 7 cm) filled with the test substrate (650 g). The cover of the black plastic box, which was punctured with small holes for ventilation, was used to prevent the earthworms from escaping. Mortality was assessed at 7 and 14 days post treatment. Ten individuals (n = 10) per replicate and four replicates (N = 4) per concentration were used for each experiment. The black plastic box was incubated in an artificial climate of 20 ± 1°C with a light: dark ratio of 16:8 h, an illumination of 600 lx and a humidity of 80–85%.

### Statistical analysis

Based on the results of the acute toxicity experiment, PoloPlus software [26] was used to calculate the sublethal concentration values (LC_10_, LC_30_, and LC_50_) and their 95% confidence intervals (CIs) for the 1^st^ instar larvae of *S*. *exigua*. Mortality was corrected using the Abbott formula. Abbott’s formula is as follows: corrected % mortality = 100 x (1-(nT/nCo)), where nT = the survivors in the treated diet and nCo = the survivors in the control [27]. The age-stage, two-sex life table parameters such as the age-stage specific survival rate (*S*_*xj*_), age-stage specific reproductive values (*V*_*xj*_), age-specific survival rate (*l*_*x*_), age-specific reproduction (*m*_*x*_), adult preoviposition period (APOP), total preoviposition period (TPOP), intrinsic growth rate (*γ*), finite growth rate (*λ*), net reproductive rate (*R*_*0*_), and average generation time (*T*) were generated and analysed by TWOSEX-MSChart software wherein the means and standard errors of the parameters were evaluated by a bootstrapping technique with 10,000 repetitions [28, 29]. The life history characteristics and population parameters of *S*. *exigua* are shown as the mean ± standard error (SE) and were further analyzed by LSD tests with SPSS 17.00 software [30]. Differences were considered significant at P < 0.05. The mortality of the treated groups was corrected using Abbott’s formula normalized to that of the control. The mortality rates (%) were transformed using arcsine √ following ANOVA and Fisher protected least significant (P ≤ 0.05).

## Results

### Toxic effects of TCAP on the larvae of *S*. *exigua*

**Fig 1:**
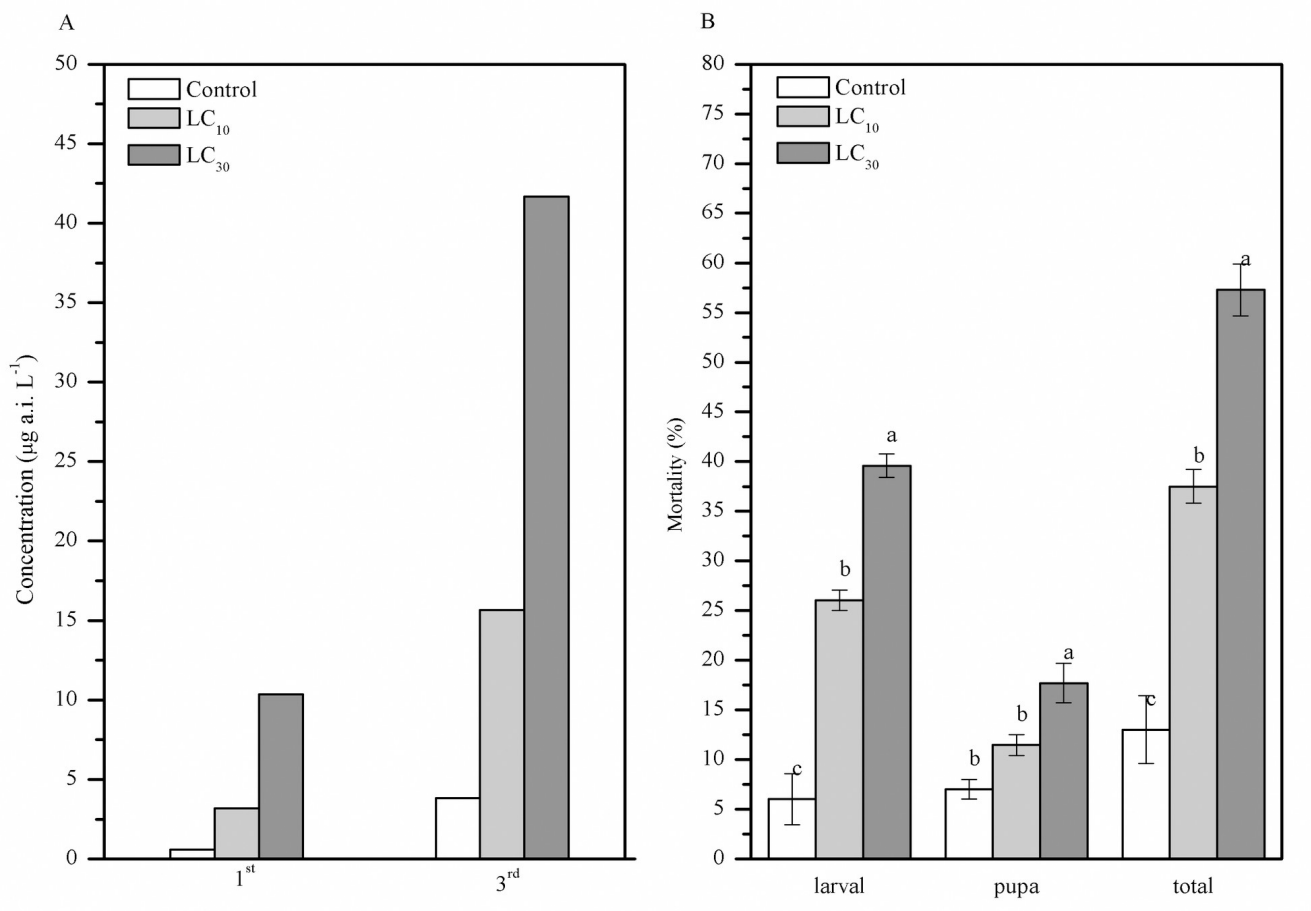
Toxicity of TCAP to *S*. *exigua*. A, LC_10_, LC_30_, and LC_50_ of the 1^st^ and 3^rd^ instar larvae of *S*. *exigua*; and B, The larval and pupal mortalities of *S*. *exigua* treated with TCAP. The bars labeled with different letters are significantly different (via Fisher’s protected least significant difference (LSD), P < 0.05).

The toxicity of TCAP on the 1^st^ and 3^rd^ instar larvae of *S*. *exigua* is shown in Fig 1A. After treatment with different concentrations of TCAP for 72 h, the LC_10_, LC_30_, and LC_50_, for the 1^st^ instar larvae were 0.578 μg a.i. L^-1^ (95% CI 0.159–1.305 μg a.i. L^-1^), 3.182 μg a.i. L^-1^ (95% CI 1.438–5.542 μg a.i. L^-1^), and 10.371 μg a.i. L^-1^ (95% CI 6.021–16.587 μg a.i. L^-1^) (Chi-squared (χ^2^) = 2.805, df = 9, P>0.05), respectively. In contrast, the LC_10_, LC_30_, and LC_50_ for the 3^rd^ instar larvae were 3.804 μg a.i. L^-1^ (95% CI 1.404–7.180 μg a.i. L^-1^), 15.648 μg a.i. L^-1^ (95% CI 8.601–23.770 μg a.i. L^-1^), and 41.672 μg a.i. L^-1^ (95% CI 28.049–58.604 μg a.i. L^-1^) (Chi-squared (χ^2^) = 4.882, df = 6, P>0.05), respectively. Compared with 3^rd^ instar larvae, 1^st^ instar larvae were more sensitive to TCAP toxicity. The mortality rate of the control group was less than 10%.

### Larval and pupal mortality

The larval and pupal mortalities of *S*. *exigua* treated with different concentrations of TCAP are shown in Fig 1B. The lowest concentration of TCAP (LC_10_) significantly inhibited larval survival, and the larval mortality increased with the TCAP concentration in a dose-dependent manner (F_2,9_ = 93.107, P<0.0001). The pupal mortality was lower than that of the larvae, indicating that the pupae were less susceptible to TCAP toxicity. The total mortality of the LC_10_ and LC_30_ reached 37.5±1.70% and 57.29±2.62% (F_2,9_ = 68.91, P<0.0001), respectively.

### Effects of TCAP on the development and reproduction of *S*. *exigua*

The development, pupal weight, longevity, and fecundity of *S*. *exigua* in different treatment groups are shown in Table 1. Compared with those of the control group, the durations of the 1^st^ and 2^nd^ instar larval and pupal stages were significantly prolonged in the LC_10_ group, while the durations of the 3^rd^, 4^th^, and 5^th^ instar larval stages were shortened. In the LC_30_ group, the entire larval stage, pupal stage, and TPOP were significantly longer than those of the control. TCAP significantly reduced the pupal weight, and this became more pronounced when *S*. *exigua* was exposed to a higher pesticide concentration (LC_30_). The fecundity markedly declined upon TCAP exposure relative to that under the control condition, but there was no significant difference between the LC_10_ and LC_30_ groups. In addition, the longevity of the female and male adults was slightly affected by TCAP; however, there was no significant difference between the three groups.

**Table 1:** Effects of TCAP on the development, pupal weight, longevity, and fecundity of *S*. *exigua*.

		Control	LC_10_	LC_30_	F(df), p
**Larval duration (days)**	**1**^**st**^	1.97±0.02c	2.92±0.03b	3.01±0.04a	F(_2,289_) = 381.764, P = 0.000
	**2**^**nd**^	2.05±0.03b	2.42±0.05a	2.41±0.06a	F(_2,266_) = 20.543, P = 0.000
	**3**^**rd**^	2.30±0.06ab	2.18±0.05b	2.38±0.07a	F(_2,234_) = 2.469, P = 0.087
	**4**^**th**^	2.49±0.06b	2.11±0.04c	2.71±0.11a	F(_2,216_) = 14.547, P = 0.000
	**5**^**th**^	4.93±0.08a	4.25±0.06b	4.46±0.09b	F(_2,200_) = 22.702, P = 0.000
**Total larval duration (days)**		13.68±0.11b	13.85±0.10b	14.77±0.22a	F(_2,200_) = 15.239, P = 0.000
**Pupal stage (days)**		6.74±0.05b	7.18±0.08a	7.29±0.09a	F(_2,184_) = 21.533, P = 0.000
**Pupal weight (g)**		95.64±0.99a	87.91±1.02b	84.22±1.77c	F(_2,184_) = 24.812, P = 0.000
**APOP (days)**		2.09±0.09a	2.13±0.10a	2.11±0.29a	F(_2,88_) = 0.025, P = 0.9750
**TPOP (days)**		25.60±0.21b	26.20±0.23b	27.33±0.49a	F(_2,88_) = 8.674, P = 0.000
**Longevity of adults (days)**	**Female**	6.26±0.20a	6.01±0.24a	6.39±0.37a	F(_2,88_) = 0.529, P = 0.591
	**Male**	5.96±0.16a	6.11±0.26a	5.87±0.33a	F(_2,93_) = 0.223, P = 0.800
**Fecundity (one female adult)**		628.56±15.29a	416.23±18.54b	400.50±21.46b	F(_2,88_) = 55.261, P = 0.000

Within a column, the means followed by different letters represent significant differences between the three groups based on the paired bootstrap test (P < 0.05). APOP: adult preoviposition period; TPOP: total preoviposition period.

### Effects of sublethal TCAP on the life table parameters of *S*. *exigua*

The life table parameters of *S*. *exigua* were substantially affected by the sublethal concentrations of TCAP. As shown in Table 2, the *γ*, *R*_*0*_, and *λ* values of the LC_10_ and LC_30_ groups were significantly lower than those of the control. In addition, sublethal levels of TCAP significantly prolonged the generation time in a dose-dependent manner.

**Table 2:** Mean (±SE) life-table parameters of *S*. *exigua* treated with TCAP.

	Intrinsic growth rate (*γ*) (d^-1^)	Net reproductive rate (*R*_*0*_) (offspring)	Finite growth rate (*λ*) (d^-1^)	Average generation time (*T*) (d)
**Control**	0.2054±0.005a	270.76±32.03a	1.23±0.013a	27.19±0.34c
**LC**_**10**_	0.1734±0.006b	125.26±19.80b	1.19±0.008b	27.79±0.21b
**LC**_**30**_	0.1485±0.008c	72.07±15.80c	1.16±0.010c	28.65±0.39a

Different letters in a column represent significant differences. In total, of 100 larvae were used for each treatment (n = 100).

**Fig 2:**
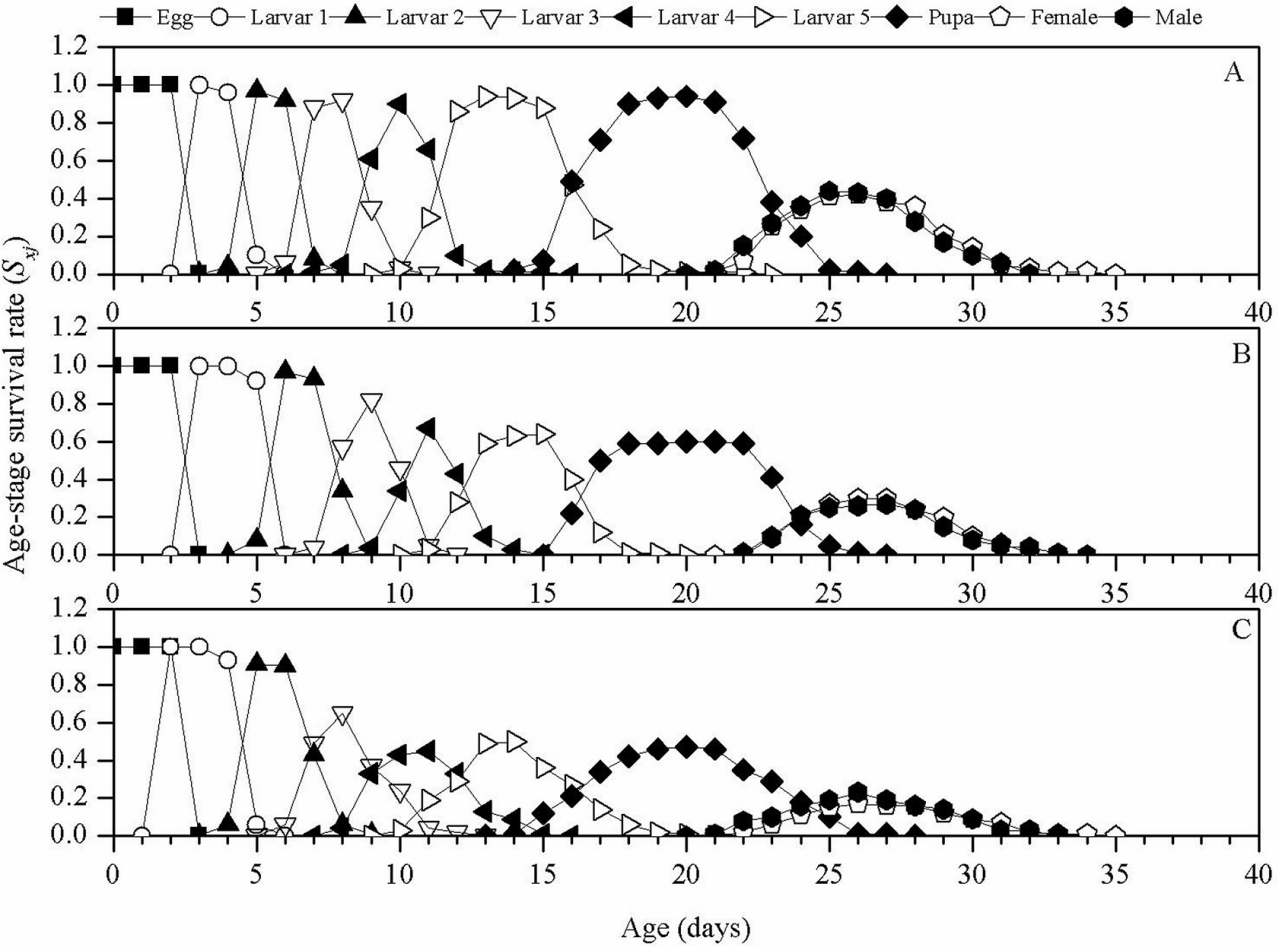
Age-stage specific survival rate (*S*_*xj*_) curves of *S*. *exigua* treated with sublethal concentrations of TCAP. A, control; B, LC_10_ group; and C, LC_30_ group.

The age-stage survival rate (*S*_*xj*_) curves (Fig 2) illustrated the survival rates of newly hatched larvae developing to the *x* and *j* stages. Due to developmental differences between individuals, there were significant overlaps between the two phases in the three treatments. Compared with those in control group, the 2^nd^ instar larvae in the LC_10_ and LC_30_ groups as well as the adults in the LC_10_ group developed later. In addition, TCAP negatively affected the *S*_*xj*_ value. Among the three groups, the *S*_*xj*_ value of the control was the highest (0.42 for the males and 0.41 for the females), while the *S*_*xj*_ values decreased with the increase of the in TCAP concentration (LC_10_ group: 0.27 for the males and 0.30 for the females; LC_30_ group: 0.20 for the males and 0.16 for the females).

**Fig 3:**
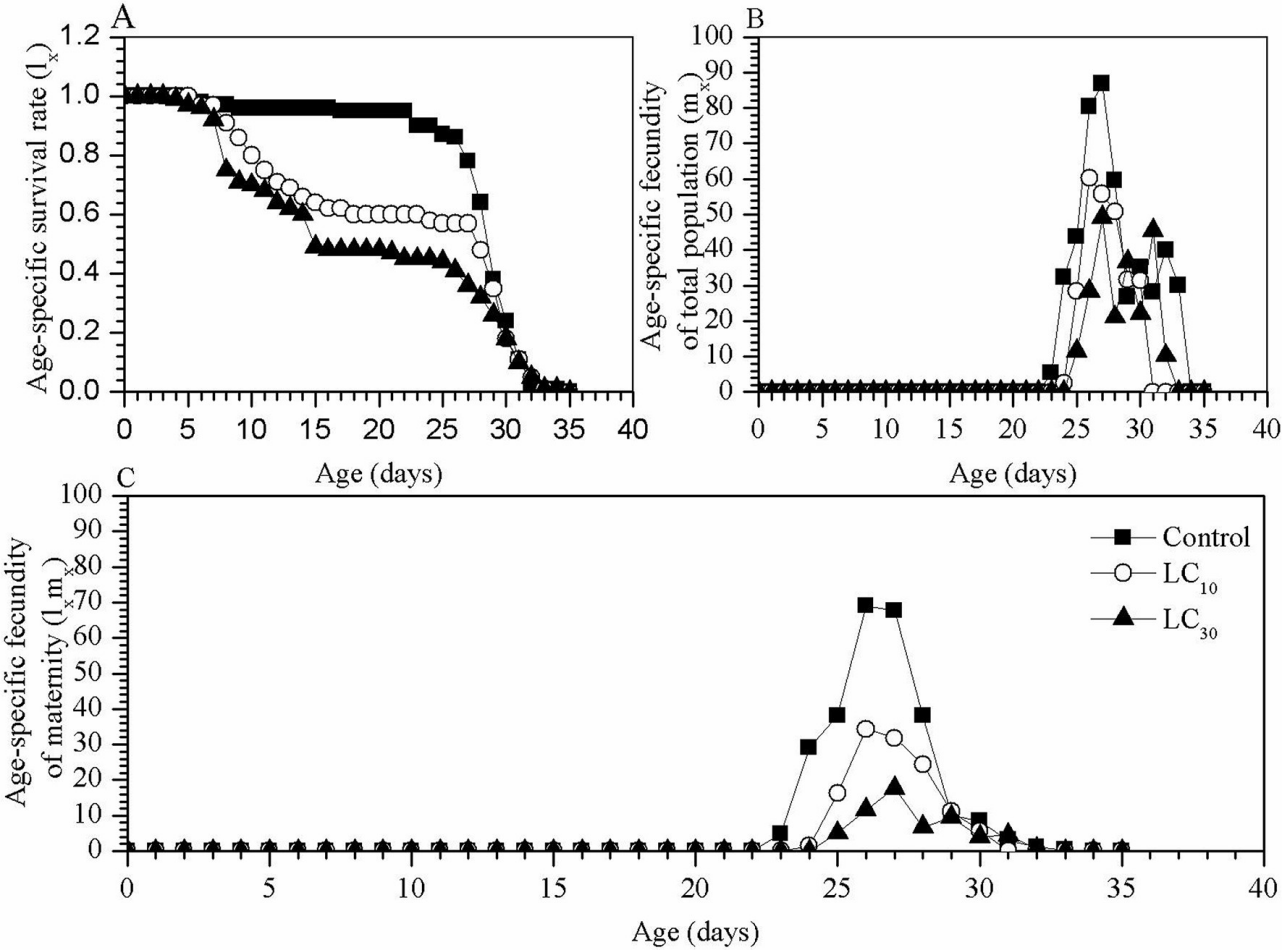
Effects of sublethal TCAP on the age-specific survival rate and population fecundity. A, age-specific survival rate curve (*l*_*x*_); B, age-specific curve of the fecundity of the total population (*m*_*x*_); and C, age-specific maternity curve (*l*_*x*_*m*_*x*_) for *S*. *exigua*.

In addition to the different developmental stages, the age-specific survival rate (*l*_*x*_) curves, which are simplified forms of *S*_*xj*_, present the survival rates of the newly hatched larvae entering age stage *x* (Fig 3A). The *l*_*x*_ of the individuals treated with TCAP significantly decreased on day 5 compared with that of the control group (Fig 3A). According to the age-specific fecundity (*m*_*x*_) curve of the total population, the starting and ending points of oviposition in the LC_10_ and LC_30_ groups were earlier than those in the control group (Fig 3B), while the spawning durations of the individuals in the TCAP treatments were shorter than those under the control conditions. Fig 3C shows the age-specific maternity (*l*_*x*_*m*_*x*_) curves, which were dependent on *l*_*x*_ and *m*_*x*_. The oviposition behaviour of the control group peaked on the 27^th^ day (69.08 eggs per adult female), while the maximum egg production in the LC_10_ and LC_30_ groups occurred on the 26^th^ and 27^th^ days, at 34.35 and 17.69 eggs, respectively.

**Fig 4:**
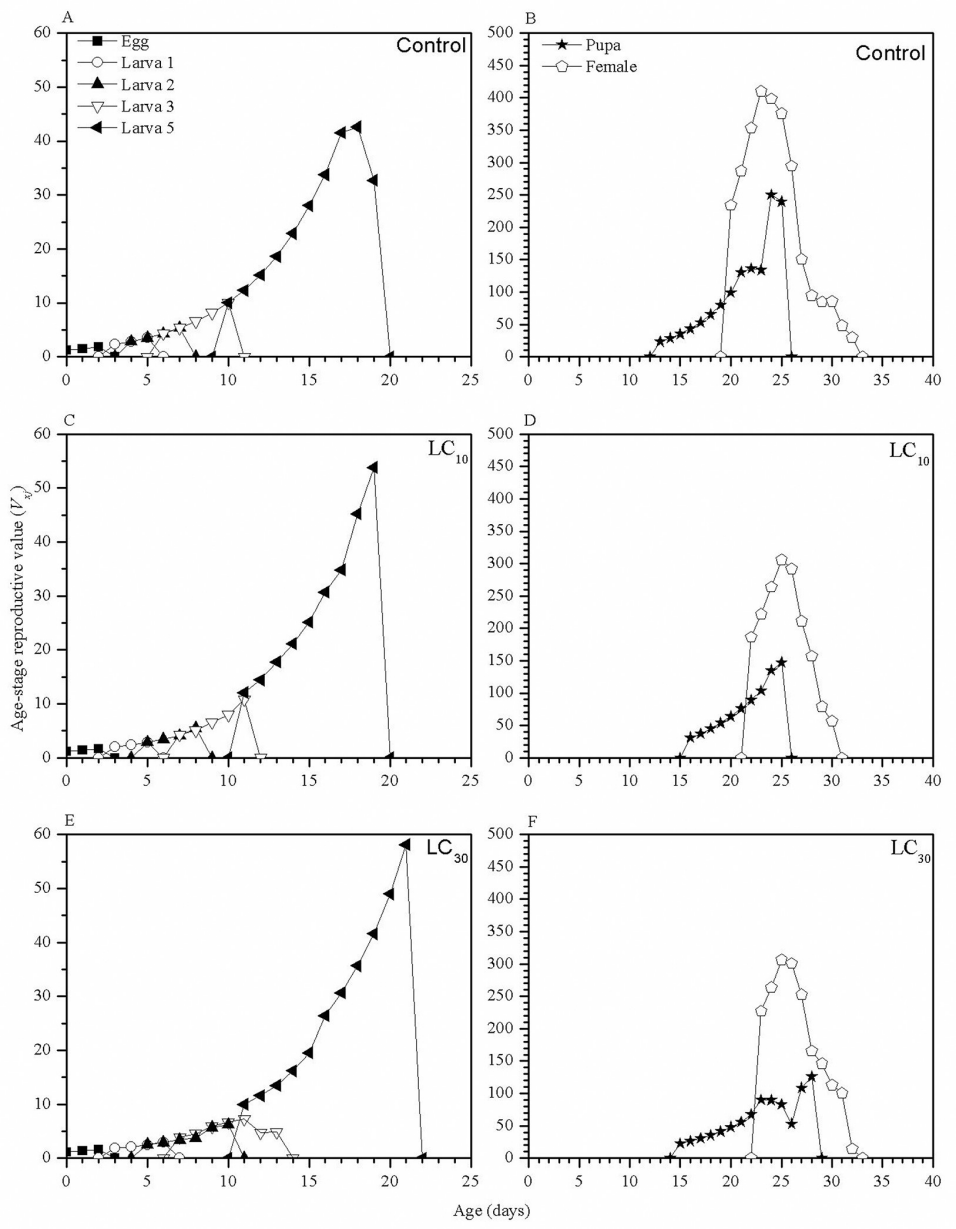
The age-stage reproduction curves (*V*_*xj*_) of *S*. *exigua*. A, the *V*_*xj*_ curves of the larvae in the control group; B, the *V*_*xj*_ curves of the pupae and adult females in the control group; C, the *V*_*xj*_ curves of the larvae in the LC_10_ group; D, the *V*_*xj*_ curves of the pupae and adult females in the LC_10_ group; E, the *V*_*xj*_ of the larvae in the LC_30_ group; and F, the *V*_*xj*_ of the pupae and adult females in the LC_30_ group.

In the larval stage, changes in age-stage reproductive values (*V*_*xj*_) between three groups were similar; however, the *V*_*xj*_ of the 5^th^ instar larvae of the LC_30_ group was higher than that of the LC_10_ and control groups (Fig 4A, 4C and 4E). For the pupal and adult stages, the peaks of *V*_*xj*_ in the LC_10_ and LC_30_ groups appeared on the 25^th^ day, with *V*_*xj*_ values of 305 and 306, respectively. The peak in the control group appeared on the 24^th^ day (*V*_*xj*_ of 410 each day) and was higher than those in the TCAP treatments groups (Fig 4B, 4D and 4F).

### Effects of TCAP on non-target organisms

The mortality of *H*. *axyridis* was significantly different between the TCAP and lambda-cyhalothrin treatments (Table 3). The results showed that the toxicity of TCAP at 2 mg L^-1^ to the larvae of *H*. *axyridis* was significantly lower than of the toxicity of lambda-cyhalothrin, which was not significantly different from that of the control (F_5, 18_ = 143.502; P < 0.0001). Regardless of the concentrations, the TCAP treatments did not trigger a significant lethal effect relative to the control (F_5, 18_ = 106.153; P < 0.0001).

**Table 3:** Acute toxicity of TCAP against the larvae and adults of *H*. *axyridis*.

Concentration of TCAP (mL L^−1^)	Mortality of larvae (% ±SE)	Mortality of adults (% ±SE)
**2 mL L**^**−1**^	52.5±4.2b	0.0±0.0b
**1 mL L**^**−1**^	35.0±2.5bc	0.0±0.0b
**0.5 mL L**^**−1**^	20.0±3.6c	0.0±0.0b
**0.25 mL L**^**−1**^	7.5±2.2d	0.0±0.0b
**Lambda-cyhalothrin (12.5 mL L**^**−1**^**)**	100.0±0.0a	27.5±4.2a
**Control**	0.0±0.0e	0.0±0.0b
**ANOVA**	F = 143.502;	F = 106.153;
	df = 5,18;	df = 5,18;
	P < 0.0001	P < 0.0001

Different letters in a column represent significant differences.

In the artificial soil test, similar to the scenario for *H*. *axyridis*, TCAP and lambda-cyhalothrin demonstrated significantly different effects on the mortality of earthworms (Table 4). The results showed that the toxicity of TCAP at 1000 mg kg^-1^ to *E*. *fetida* after 7 days and 14 days was significantly lower than that of lambda-cyhalothrin, and there was no significant difference compared with the control levels.

**Table 4:** Toxicity of TCAP against *E*. *fetida* earthworms.

Treatment and concentration (mg kg^−1^)	7th day (mortality% ±SE)	14th day (mortality% ±SE)
**TCAP-1000 mg kg**^**−1**^	2.5±2.2c	5.0±2.5b
**TCAP-500 mg kg**^**−1**^	0.0±0.0c	0.0±0.0b
**TCAP-250 mg kg**^**−1**^	0.0±0.0c	0.0±0.0b
**Lambda-cyhalothrin-32 mg kg**^**−1**^	100.0±0.0a	100.0±0.0a
**Lambda-cyhalothrin-16 mg kg**^**−1**^	95.0±2.5ab	100.0±0.0a
**Lambda-cyhalothrin-8 mg kg**^**−1**^	87.5±2.2b	97.5±2.2a
**Control**	0.0±0.0c	0.0±0.0b
**ANOVA**	F = 237.033;	F = 301.306;
	df = 6,21	df = 6,21
	P < 0.0001	P < 0.0001

Different letters in a column represent significant differences.

## Discussion

Insecticides are generally unevenly distributed and degrade when applied in the field, which enhances the probability of insect exposure to low concentrations of insecticides. Therefore, studying the sublethal effects of insecticides on target insects could improve the rational utilization of pesticides [31–33]. As a newly developed diamide insecticide, TCAP is generally used to control lepidopteran pests, such as *P*. *xylostella* [34]. By reviewing the literature, we found that *S*. *exigua* larvae were more sensitive to TCAP (LC_50_, 10.371 μg L^-1^) than to other insecticides, such as chlorantraniliprole (LC_50_, 35 μg L^-1^), cyanamide (LC_50_, 92 μg L^-1^), chlorpyrifos (LC_50_, 2401 μg L^-1^), and emamectin benzoate (LC_50_, 537 μg L^-1^), avermectin (LC_50_, 2687 μg L^-1^), hexaflumuron (LC_50_, 7384 μg L^-1^), and methoxyfenozide (LC_50_, 2167 μg L^-1^) [2]. Our results showed that the fecundity and longevity of *S*. *exigua* larvae and adults were significantly decreased in the LC_10_ and LC_30_ TCAP groups, which was consistent with the finding of Lai and Su that sublethal concentrations of chlorantraniliprole suppressed *S*. *exigua* fecundity [35], indicating that TCAP is an effective alternative tool for *S*. *exigua* management.

Studies have shown that sublethal concentrations of some insecticides induce fecundity-stimulating effects and result in a resurgence of the insect pest population. For example, sublethal doses of imidacloprid and azadirachtin stimulated the reproduction of green peach aphids, and sublethal doses of triazofos and pyrethroids contributed to the enhanced fecundity of brown planthoppers [36, 37]. In contrast, this study revealed that the fecundity of *S*. *exigua* treated with sublethal concentrations of TCAP was significantly reduced compared to the control level. It seems that the LC_10_ of TCAP disrupted the energy homeostasis and prolonged the larval stage of *S*. *exigua*, contributing to larvae shifting energy from growth to detoxification metabolism [38]. Likewise, the LC_30_ of TCAP retarded the growth of *S*. *exigua* with a prolonged TPOP. These results illustrated that a sublethal concentration of TCAP did not induce resurgence in the *S*. *exigua* population.

The age-stage, two-sex life table theory can be to evaluate of the effect of insecticides on insect populations. The life table parameters can be used to assess the sublethal effects of insecticides at the population level [39, 40]. The population parameters, such as *γ*, *λ*, and *R*_*0*,_ substantially declined in the LC_10_ and LC_30_ groups compared with those in the control group, suggesting that sublethal TCAP concentrations inhibited the growth rate and reproduction of *S*. *exigua*, which was in agreement with the report from Lanka et al. [41]. According to the *S*_*xj*_ and *l*_*x*_ curves, sublethal TCAP levels reduced the survival rate of the *S*. *exigua* population. Furthermore, TCAP inhibited reproductive efficiency, as concluded from the *V*_*xj*_ and *m*_*x*_ curves. These results strongly indicated a reduction in the number of *S*. *exigua* population. Other studies have also shown that the sublethal effects of multiple insecticides adversely affected the life table parameters of pests and reduced the number of F_1_ offspring [23, 42].

For pesticide application in agrosystems, it is of parallel importance to both determine the insecticide efficacy against the target organisms and assess its hazards to nontarget organisms. In this study, the effect of TCAP on the mortality of *H*. *axyridis*, which is a natural enemy of *S*. *exigua*, was determined. TCAP did not show any toxicity toward the adults of *H*. *axyridis* (Table 3). Similarly, chlorantraniliprole has been proven to be safe for *Orius laevigatus*, and sunflower EFN contaminated with chlorantraniliprole caused no lethal effects when consumed by *Lysiphlebus testaceipes* adults [43, 44]. We also found that when exposed to TCAP, the mortality of larvae of *H*. *axyridis* increased with the TCAP concentrations and exhibited a dose-response relationship (Table 3). This result is partially attributed to the stronger xenobiotic tolerance and defense capacity of adults than of larvae. Nevertheless, for the larvae of *H*. *axyridis* exposed to 0.25 mL L^-1^–0.5 mL L^-1^ TCAP, the average mortality was below 20%, which was in line with the finding that chlorantraniliprole was less toxic to *Podisus nigrispinus* and *Supputius cincticeps* nymphs, resulting in mortality rates for both species of less than 10% when exposed to 10x the recommended field rates of chlorantraniliprole for 72 h [45]. Additionally, chlorantraniliprole caused less than 25% mortality in *Macrolophus pygmaeus* and was classified as harmless according to the International Organization for Biological Control rating scheme [46], its environmental stress on natural enenmies could not be disregarded. For example, the chlorantraniliprole applied at recommended field rates for the control of *Chloridea virescens* caused high mortality in adult predator *Hippodamia convergens* [47].

Pesticides are directly applied to soil for controlling soil borne pests or deposited in soil as runoff from foliar applications [48]. The concentrations applied to control pests are high enough to affect non target organisms and constitute potential hazards to the structure and functioning of terrestrial ecosystems [49]. Earthworms as “ecological engineers” play pivotal roles in maintaining soil physicochemical structure, facilitating aeration, energy flow, nutrient cycling as well as enhancing soil fertility [50]. Due to their close contact and high sensitivity to toxicants, they have been identified as bio-indicators for early warning of soil pollution [51]. Earlier reports have revealed that pesticides may exert detrimental effects on earthworm at all organisation levels from sub-individual level to community level [52, 53] and the chemicals should be subjected to environmental risk assessment before registration or commercial use. Consequently, we employed the standardised test species *E*. *fetida* to evaluate the non-target and ecological toxicity of TCAP.

The toxicity of pesticides to earthworms depends on a variety of factors, such as the physicochemical properties of soil, exposure concentration and duration, and degradation dynamics in soil etc [50, 54, 55]. Vasantha-Srinivasan et al. [5] compared the toxicity of temefos and monocrotofos to *E*. *fetida* and estimated that the LC_50_ values of temefos and monocrotofos were 3.8 and 5.3 mg kg^-1^, respectively. Wang et al. [55] reported that clothianidin was the most toxic to the earthworm *E*. *fetida* among nine pesticides, with LC_50_ values of 7.44 mg kg ^-1^ and 6.06 mg kg ^-1^ at day 7 and day 14, respectively. According to Mali [56], the suggested standard of toxicity is LC_50_ <1 mg kg^-1^ for highly toxic pesticides, 1–10 mg kg^-1^ for moderately toxic pesticides, and >10 mg kg^-1^ for mildly toxic pesticides. Hence, the toxicities of temefos, monocrotofos and clothianidin were designated moderate. However, the other 8 pesticides were considered mildly toxic, with LC_50_ values well beyond 100 mg kg^-1^ [55]. Likewise, Wang et al. [54] examined the toxic effects of 24 insecticides from distinct classes on *E*. *fetida*. Among them, the four pyrethroid insecticides tested were the least toxic, with LC_50_ values higher than 1000 mg kg^-1^. Herein, the toxicity of TCAP to *E*. *fetida* was tested, and even a concentration of 1000 mg kg^-1^ did not induce significant death in the earthworms. Therefore, this pesticide may have low toxicity to terrestrial ecosystems and living organisms.

## Conclusions

In conclusion, TCAP displayed a lower toxicity to *S*. *exigua* than other insecticides. Sublethal concentrations of TCAP (LC_10_ and LC_30_) increased the larval and pupal durations, prolonged the average generation time and reduced the survival rate, longevity, and fecundity of *S*. *exigua*. Notably, no population resurgence occurred. TCAP was non-toxic to *H*. *axyridis* ladybugs and *E*. *fetida*, suggesting that it could be environmentally friendly for natural enemies and soil invertebrates, which is of great importance for ecotoxicological and environmental fate studies focusing on TCAP. Overall, the present study provides a reference for the proper and safe utilization of TCAP to suppress *S*. *exigua* populations in the field.

Further research should include ecotoxicological risk assessments, the interaction between insecticides and natural enemies and the sublethal side effects of insecticides on other predators.
